# Progression of Quantum Dots Confined Polymeric Systems for Sensorics

**DOI:** 10.3390/polym15020405

**Published:** 2023-01-12

**Authors:** Ranjana Choudhary Ahirwar, Swati Mehra, Sanjeev Machindra Reddy, Hassan Abbas Alshamsi, Aseel A. Kadhem, Smita Badur Karmankar, Alka Sharma, Poushali

**Affiliations:** 1 Department of Chemistry, IPS Academy, Institute of Engineering and Science, Indore 452012, India; 2 Department of Chemistry, Gramin (Arts, Commerce & Science) Mahavidyalaya, Vasantnagar K. Tal. Mukhed Dist., Nanded 431715, India; 3 Department of Chemistry, College of Education, University of Al-Qadisiyah, Diwaniya 58002, Iraq; 4 Department of Chemistry, Iraqi Ministry of Education/Wasit Education Directorate, Al Khajia District, District 327, Alley 26, House No. 6, Wasit 52001, Iraq; 5 Bar-Ilan Institute for Nanotechnology and Advanced Materials, Ramat Gan 5290002, Israel

**Keywords:** fluorescence, quantum dots, chemical stability, QDs/polymer hybrids, sensors

## Abstract

The substantial fluorescence (FL) capabilities, exceptional photophysical qualities, and long-term colloidal stability of quantum dots (QDs) have aroused a lot of interest in recent years. QDs have strong and wide optical absorption, good chemical stability, quick transfer characteristics, and facile customization. Adding polymeric materials to QDs improves their effectiveness. QDs/polymer hybrids have implications in sensors, photonics, transistors, pharmaceutical transport, and other domains. There are a great number of review articles available online discussing the creation of CDs and their many uses. There are certain review papers that can be found online that describe the creation of composites as well as their many different uses. For QDs/polymer hybrids, the emission spectra were nearly equal to those of QDs, indicating that the optical characteristics of QDs were substantially preserved. They performed well as biochemical and biophysical detectors/sensors for a variety of targets because of their FL quenching efficacy. This article concludes by discussing the difficulties that still need to be overcome as well as the outlook for the future of QDs/polymer hybrids.

## 1. Introduction

In recent decades, continuous advances in the synthesis and production of novel materials have further boosted the development of nanoscience and technology [1,2,3,4,5,6,7]. Quantum dots (QDs) are a relatively novel phenomenon in the world of the 0D nanomaterials’ family of minerals. Particles with sizes smaller than the exciton Bohr radius are called QDs [8]. Xu et al. (2004) discovered that luminous carbon nanomaterials may be produced by the purification of SWCNTs [9]. They have been identified as separate, quasi-spherical, clustered domains of many carbon atoms with a dimension of less than 10 nm. Then, in 2006, Sun et al. developed identical fluorescent (FL) carbon nanoparticles, which they dubbed carbon quantum dots (CDs), both dispersed in a solvent and as an extract in a solid form, both of which were fluorescent (FL) [10]. As a result, several researches have been carried out to investigate CDs from the perspectives of synthesis, characterization, and application [11,12]. When C atoms in CDs hybridize with surface ligands, the state of hybridization in the core of the CD is sp^2^. Surface ligands are derived from the basic materials themselves. The oxygenated groups found in surface ligands are often carbonyls, carboxylic acids, hydroxyls, and epoxy groups to name a few examples. In addition, carbonized polymer dots (CPDs) are gaining popularity, which are a novel type of CDs with a polymer/carbon hybrid structure that has begun to attract attention [13,14]. Thus far, the optical qualities of these QDs have been the most actively pursued field of research for them [15]. Photoluminescence (PL) is one of the most prevalent characteristics of QDs that researchers are looking for. One type of PL is excitation wavelength-dependent, whereas the other type is excitation wavelength-independent. PL is categorized into two separate types. PDs and CPDs are categorized as CD polymers by certain researchers, despite the fact that they are classed as CD polymers by others. The precursors and synthesis procedures for both PDs/CPDs and CDs, on the other hand, are almost similar. As a unique representative of the intriguing zero-dimensional carbon nanomaterials family, QDs have piqued the public’s curiosity due to their unique physical, chemical, and optical properties. QDs are now being studied further [16]. Oxygenated groups such as hydroxyls, carboxylic acids, carbonyls, and epoxy groups are the most common types of surface functional groups found on surfaces [17]. Additionally, due to their biocompatibility, CDs have been investigated as a potential green alternative to heavy metal-containing conventional semiconductor QDs. Simple preparation, a wide range of precursors, strong hydrophilicity, great sensitivity and selectivity, adjustable surface and fluorescence characteristics, minimal cytotoxicity, outstanding electron transport capability, and chemical inertness are just some of QDs’ many benefits [18,19,20,21].

The fact that several studies of quantum dots’ PL behavior and applications in a range of disciplines have previously been published demonstrates that there is a need for a single, more concentrated assessment of QDs/polymer composite sensors for sensor applications [22,23,24]. Currently, the merging of QDs with other functional molecules/materials to make composites has piqued the curiosity of researchers who are looking into the possibility of doing so [25]. Composites have the potential to significantly alleviate the inadequacies of pure QDs while also expanding their multifunctional uses. Because of the abundance of surface functional groups on QDs, they may readily be combined with other materials to make composites [26]. Because of the tiny size and vast number of surface functional groups on CDs, they have a strong contact with polymers, making it feasible to achieve homogenous dispersion inside the polymers [27]. Remarkable properties of these composites include strong optical absorptivity, high aqueous solubility, good functionalization, photobleaching resistance, excellent chemical stability, low toxicity, and high biocompatibility, among other characteristics. Improved flexibility, strength, durability, and hydrophobicity are other characteristics of CD/polymer composites [28].

This overview is necessary in order to comprehend the current advancement of CDs/polymer composites in the sensor domain, as well as certain critical features of the technology. The most important elements of CDs/polymer composites, such as their precursors, synthesis processes, optical properties, coating characteristics, and sensing applications, are discussed in detail. We are solely interested in the QDs synthesis methods that were used prior to the preparation of QDs/polymer composites in this review. We address the optical properties of QDs/polymer composites, as well as various physicochemical and coating parameters that have an impact on the optical properties. In the next section, we particularly separate QDs/polymer composites’ sensing applications into different types of sensing categories. The remaining challenges and future perspectives for CDs/polymer composites are discussed in order to improve their functionality, including the investigation of new green and inexpensive precursors, the one-step synthesis method, the heteroatom doping effect, and the development of new sensing techniques.

## 2. Preparation Methods of QDs

The synthetic approaches of the QDs can be divided into two main groups: the top-down approach and the bottom-up approach. The top-down process is achieved by cleaving or disintegrating carbonaceous materials via a chemical, physical, or electrochemical technique. The bottom-up method can be accomplished through the carbonization of small molecules, the pyrolysis of small molecules, or the chemical fusion of organic molecules in a step-by-step fashion. The physicochemical characteristics of the nanoparticles are determined by the synthesis technique as well as the precursors. These properties include the size of the nanoparticles, surface properties, oxygen or nitrogen content, suitability with various solvents, and colloidal stability. The top-down approach has a number of benefits, including large-scale production, and an abundance of raw materials. In addition, synthesis accomplished through the use of this approach typically results in the presence of oxygen functional groups at the edges, which boosts the solubility of the nanoparticles as well as the surface passivation. However, the top-down method has a few drawbacks, such as a low yield, the need for specialized instrumentation, the potential for damage to the aromatic carbon structure, and the nonselective chemical cleavage procedure. Because of these limitations, this technique does not provide good control over the shape and size distribution of the nanoparticles [29,30]. On the other hand, the bottom-up method offers a variety of possibilities to regulate the morphology, size, and, therefore, the attributes of the QDs [31]. These methods can be effective, but they often demand the use of organic precursors that are either hard to obtain or involve a complex synthesis procedure [32].

### 2.1. Top-Down Method

There are different types of synthesis methods in this approach including arc discharge, laser ablation, the electrochemical synthesis method, etc. According to Arora and Sharma [33], arc discharge [34] is a method that can be used to restructure the carbon atoms that have been decomposed from the bulk precursors material in the anodic electrode of an enclosed reactor. This technique is driven by the gas plasma that is produced in the reactor. In order to generate plasma with a high energy output, the temperature inside the reactor can rise to as high as 4000 K when it is subjected to an electric current. At the cathode, QDs are formed through the assembly of carbon vapor. This method of manufacturing CDs through arc discharge was first developed in 2004 [9]. Dey et al. reported boron-doped graphene quantum dots (GQDs) prepared by the arc discharge method [35]. The authors reported chemical scissoring of B-doped graphene generated by arc discharge in the gas phase followed by the synthesis of GQDs. These GQDs showed strong blue emission and excitation-independent emission behavior. Using femtosecond laser ablation and the sonication-assisted liquid exfoliation method, Xu and coworkers developed a simple and fast method for the synthesis of high-quality MoS2 and WS2 QDs [36]. The prepared samples had a few layers and an average size of 3.7 nm and 2.1 nm and showed blue–green emission under UV irradiation. Cu-doped ZnS QDs were synthesized through femtosecond laser ablation of a bulk ZnS:Cu target carried out in deionized water by Zheng et al. [37]. The obtained QDs have good colloidal stability, as well as narrow and symmetric Cu-related emission, similar to the common blue emission from bulk ZnS:Cu. By varying the laser fluence, the mean size of the QDs varies from 2.1 to 4.0 nm, which tends to result in a redshift of the emission peak. By oxidizing a graphitic column electrode at 3 V against a saturated calomel electrode while employing a Pt wire counter electrode in 0.1 m NaH_2_PO_4_ aqueous solution, Zhao et al. was able to fabricate CDs electrochemically [38]. As oxidation time increased, the electrolyte solution turned from colorless to yellow, followed by dark brown. CDs obtained from the smaller two MW fractions had sizes of (1.9 ± 0.3) nm and (3.2 ± 0.5) nm. HRTEM studies suggested that the CDs were graphitic in nature with a lattice spacing of 3.28 Å, and the PL studies revealed size-dependent emission behavior.

### 2.2. Bottom-Up Method

**Figure 1:**
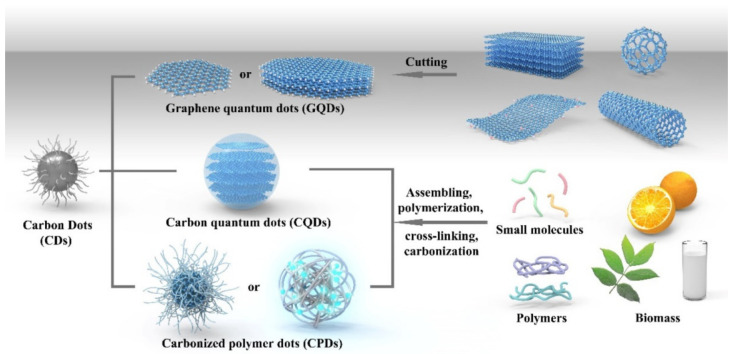
Different fabrication approaches of GQDs, CQDs, and CPDs. Reproduced with permission from ref. [41].

High-temperature pyrolysis or carbonization of the precursors has been widely used for several decades. This approach results in irreversible alterations to both the physical phase and chemical characteristics. Bourlinos and colleagues utilized a one-step thermal decomposition of low-temperature-melting molecular precursors to generate hydrophilic or organophilic surface-passivated QDs [39]. This process is extremely desirable due to the fact that it directly results in surface-passivated QDs that have precisely engineered surface properties. Furthermore, by selecting an appropriate carbon source and surface modifier, it is possible to have improved control over the geometry and physiochemical characteristics of the QDs. CDs were extracted from the natural gas combustion soot by Tian and his coworkers [40]. According to observations from TEM images, the resultant particles had an average diameter of 4.8 ± 0.6 nm, and the crystalline lattices were comparable with graphitic carbons. Furthermore, FTIR and ^13^C NMR spectroscopy verified the existence of sp^2^ carbons in the form of aryl and carboxylic/carbonyl moieties. A simple and inexpensive pyrolysis of citric acid yielded luminescent GQDs with abundant sp^2^ clusters. Changing the carbonization quantity and then dispersing the result in alkaline media led to the synthesis of GQDs [32]. The synthesis of nanomaterials using microwave techniques has several benefits, a rapid reaction duration, fast and uniform heating, and enhanced yield and purity. Gant et al. investigated the possibility of producing fluorescent QDs by microwave-assisted synthesis by employing folic acid as both the carbon and nitrogen precursor. In brief, folic acid was dissolved in diethylene glycol and heated for 40 s at 750 W in a microoven. The obtained particles revealed an average size of 4.51 nm, water solubility, excellent biocompatibility, blue emission, and 18.9% QY at an excitation wavelength of 360 nm. Figure 1 shows that GQDs, CDs, and carbonized polymer dots (CPDs) are different according to their distinct formation mechanisms, their micro- and nanostructures, and their characteristics. They can also be associated together by changing the graphene layer and the amount of carbonization.

## 3. Fluorescence Origin, Quenching/Restoration Mechanism, and Different Types of Fluorescence Sensors

**Figure 2:**
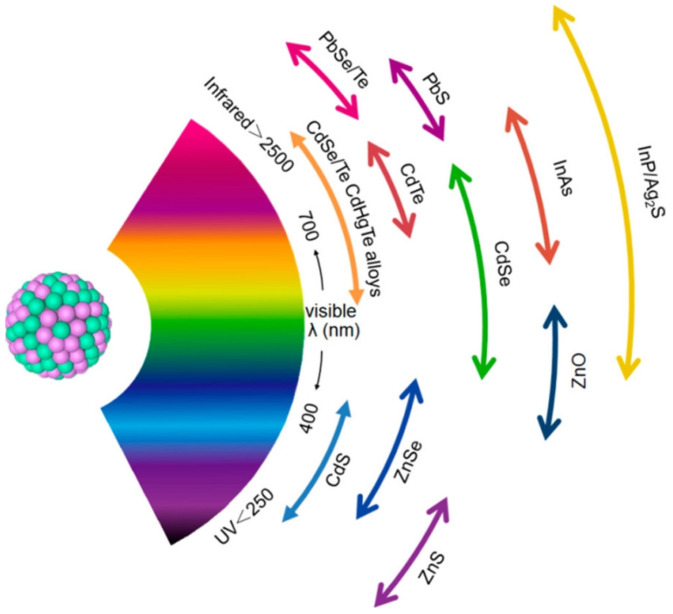
Pictorial representation of core material of various QDs scaled according to their emission wavelength over the spectrum. Reproduced with permission from ref. [50].

The luminescence mechanism is vital for guiding the synthesis of QDs with tunable fluorescence emission. Despite numerous studies, the universal fluorescence origin of QDs remains a mystery and a source of contention. QDs developed using a variety of substances and methods typically contain diverse, intricate components and architectures. In other words, QDs created using diverse synthetic methods, precursors, and post-treatments have dissimilar optical properties, showing that CDs are a more complex system than anticipated. Therefore, it is inappropriate to construct a unified theory by comparing the attributes of QDs described in diverse literature publications. The surface state [42,43], quantum confinement effect [44,45], and molecular fluorescence [46,47] are presently the most widely accepted luminescence origin mechanisms. The surface state mechanism includes the degree of surface oxidation and surface functional groups. Luo et al. synthesized CQDs using a hydrothermal technique using hydroquinone and ethylenediamine and separated them using silica gel chromatography to acquire blue, green, and yellow emissive CQDs fractions [48]. It was revealed that their surface state was primarily responsible for their multicolor emissive photoluminescence. The photoluminescence emissions of these QDs were strongly related to the functionalities (C=N) present at the surface. Because of the increasing C=N content at the surface of the QDs, the band gap reduced, which caused red shift of the emission peak. By altering the size and composition, the emission color of QDs can be altered [49]. The emission wavelength of QDs can be tuned to cover the ultraviolet (UV), visible, and near-infrared (NIR) regions by selecting a suitable core material with a desirable band gap and tuning the size of the QDs (Figure 2).

QDs have been extensively utilized in the fields of nano-sensors, bio-imaging, and optoelectronics, as key components in the assembly of functional nanomaterials, due to their flexible processibility and distinct size-dependent characteristics. Self-aggregation by means of noncovalent interactions including H-bonding interaction, π–π interaction, electrostatic forces, van der Waals forces, etc., has been demonstrated to be an effective mechanism for the assembly of nanoparticles, and the noncovalent interactions frequently bestow reversibility upon the aggregation process [51]. The aggregation-induced fluorescence-quenching-based sensor probe for the detection of glucose was reported by Das et al. [11]. In another work, these authors demonstrated the fluorescence quenching mechanism for sensing Cr(VI) was an inner filter effect (IFE) [52]. The IFE phenomenon occurs when the quencher’s absorption band has a high spectral overlap with the excitation and/or emission wavelengths of a fluorescent agent. The introduction of ascorbic acid to the JB-CD/Cr(VI) sensor system resulted in the reduction of Cr(VI) to Cr(III)/lower valence Cr species, hence eliminating the IFE effect and restoring the fluorescence of the JB-CD system. Additionally, fluorescence recovery may be the result of chelation, where the surface functional groups of JB-CDs are susceptible to binding Cr(VI) in the presence of ascorbic acid. Fluorescence quenching is also ascribed to an energy transfer mechanism or an electron transfer mechanism. Commonly, the energy gap between the LUMO of the donor and acceptor is the driving factor of electron transfer [53]. Therefore, if the most important mechanism for quenching is electron transfer, then the compounds with the lowest LUMO (such as nitro compounds) should have the greatest quenching.

Due to their high sensitivity, high specificity, immunity to light scattering, and ease of operation, fluorescent sensors are commonly used for the detection of biomolecules or metal ions. Due to the significant characteristics that set them apart from other approaches, fluorescence methods hold a special place in biophysical research as well as in a wide variety of applications in imaging and sensing. The greatest absolute sensitivity, which in certain unique studies approached the limit of single molecules, is one example. However, the picomolar detection range may commonly be attained even in routine tests. This is especially important if the analyte is only present in minute quantities, and the time- and money-intensive enrichment processes must be avoided. Obtaining photographs with a high level of detail requires having a spatial resolution that is quite high. In conventional microscopy, this is restricted to a fraction of the wavelength of the light being used; however, by exceeding the restriction imposed by light diffraction, the molecular scale may now be observed. These methods make it possible to acquire the quickest reaction that can occur on the timescale of the fluorescence lifetime, and they can detect events on a timescale that is as short as 10^−8^–10^−12^ s. The transition from bright to dark, or vice versa, in the fluorescence signal may be easily viewed and recorded as a change in the intensity of the fluorescence at a single wavelength. It is possible that great spectral resolution is not required for these studies because the fluorescence spectra of organic dyes are very wide (often with half-widths ranging from 50 to 70 nm). Fluorescence quenching or enhancement is often employed in a variety of sensing and imaging systems as the reporter signal [54]. In vivo imaging benefits greatly from the use of fluorescent proteins and genetically encoded sensors due to their ability to selectively label and illuminate just the cells or organs of interest. When compared to large molecules, small molecules have the advantage of not requiring genetic engineering of the target animal genome for in vivo imaging. However, ensuring that small molecule probes are delivered to the site of interest remains a major challenge due to the many obstacles faced in transfer and in accurately adjusting pharmacodynamic parameters. In terms of fluorescence-based equipment, the filter-based fluorometer is the most conventional form. While some of this instrument’s parts are comparable to those of a spectrometer, others, such as the excitation and emission wavelength selectors, are replaced with optical filters. Filter-based fluorometers often have a smaller footprint, lower cost, and are easier to set up since they do not require the use of monochromators for excitation and emission wavelength selection, as is the case with more traditional spectrometers. Therefore, the filter-based fluorometer arrangement is commonly used for portable fluorescence sensing applications due to its simplicity and portability. Selectivity is restricted to a small number of filters for both excitation and emission wavelengths due to the one-way nature of optical filters. In most cases, filter-based fluorometers are the best choice when periodic quantitative measurement of a single analyte is required. Piruska et al. reported on a comparison of autofluorescence across a variety of materials [55]. The results of this investigation showed that autofluorescence emission was maximum when stimulated by blue light (403 nm). Under 403 nm, PDMS has around four times the autofluorescence of borosilicate glass. When compared to COC and PC, PMMA showed 41 times stronger tenacity, while COC showed six times higher. Autofluorescence is a source of noise in biological sensing applications; however, thermoplastic materials have been employed because they are robust against temperature and pressure. An appropriate choice of excitation light is crucial for increasing the sensitivity, selectivity, and other aspects of the fluorescence sensing system. Many different types of excitation light sources are used in diverse contexts, and each has its own set of benefits and drawbacks. For the least amount of disruption to the fluorescence from emission, it is best if the excitation light has a small spectral linewidth. A lamp, an LED, and a light-amplification-by-stimulated-emission-of-radiation (LASER) light source are each detailed here along with their unique qualities (laser). Another type of sensor called a colorimetric sensor is an optical sensor that changes color in response to external stimuli. A stimulus can be any change in the physical or chemical environment. Therefore, the design of the specific colorimetric sensor is determined by the change in an environmental quality that must be detected. In colorimetric sensors, the large shift is usually noticed corresponding to a distinct color change detected by the naked eye.

## 4. Encapsulation of QDs inside Polymer Matrix and Their Composites

Polymer matrices are unique owing to the fact that they are simple to process, have a low specific gravity and interesting physical features, and are ductile. However, in comparison to ceramics and metals, their modulus values are significantly lower, which is a severe drawback. Therefore, the technique that was most commonly taken was to incorporate foreign particles that were relatively high in elastic qualities in order to increase their mechanical properties. These foreign particles are generally called fillers and they can be divided into two categories according to their mode of application or serviceability. In general, micron-sized fillers are utilized to improve the properties of polymer composites ever since the early stages of polymer research. However, the nanosized inclusions made them significantly more effective in practically all uses. In this perspective, QDs are one of the most advanced and high-quality nanomaterials; hence, a significant amount of research for the incorporation of QDs into polymer matrices is of the utmost relevance [56,57]. The reinforcing behavior of QDs in polymer matrices has been the point of discussion and reported by a number of different researchers [58,59]. In addition, considering that QDs are nanoparticles at a nanoscale level and have high surface energy, the issue of stability is a significant problem. These nanoparticles exhibit a high number of surface charges, which are balanced by polymer chains or polymer pendent groups. These electrical charge balances may lead to improved stability within polymer matrices. When QDs are encapsulated within polymer matrices, there are two primary options for anchoring. Either chemical attachments in the form of covalent or ionic interactions and physical anchoring can be considered as these two types of forces. Adsorption of polymer chains onto QDs particles is referred to as “physical anchoring”. These QDs can improve the polymer flexibility, strength, durability, and hydrophobicity, among other desirable characteristics. When QDs are mixed or entrapped into polymer matrices, polymer chains are actually physisorped on to it. The tightly bound polymer should be compatible with the QDs’ surface polarity, otherwise there will be a strong chance of phase out. For polar–polar compatibility, in most cases, hydrophilic polymer systems provide a better result. Moreover, the flow behavior is restricted for polymer systems when QDs are physically blended with the polymer phases. This is the cause of chain–polymer interaction, and anchoring among the surface polarities of QDs are polymer functionalities [60]. In other words, the concentration of QDs in a polymer system also improved their delayed rupture and thixotropy. These are not only prominent in a physically mixed system but also in the chemically/covalently cross-linked systems [61].

Chowdhury et al. reported the synthesis of a nanocomposite hydrogel film composed of chitosan and CDs that has remarkable features. This film was soft but robust. The CDs that were used to make the nanocomposite derived from tea. The authors demonstrated that appropriate electrostatic interaction between the positive charge on the chitosan and the negative charge on the CDs was the fundamental requirement for the fabrication of such hydrogel films. In comparison to pure chitosan hydrogel films, nanocomposite hybrid films were able to achieve much better results in terms of UV–visible blocking ability, thermal stability, and mechanical strength attributed to the electrostatic interactions. For the nanocomposite hybrid films, the percent transmittance in the 300–600 nm wavelength region was reduced by up to 20%, tensile strength increased significantly to 18.6 MPa, and the change in contact angles was from 64.95° (pure chitosan film) to 88.75°, suggesting that the hydrophobicity of the nanocomposite hydrogel films was improved.

**Figure 3:**
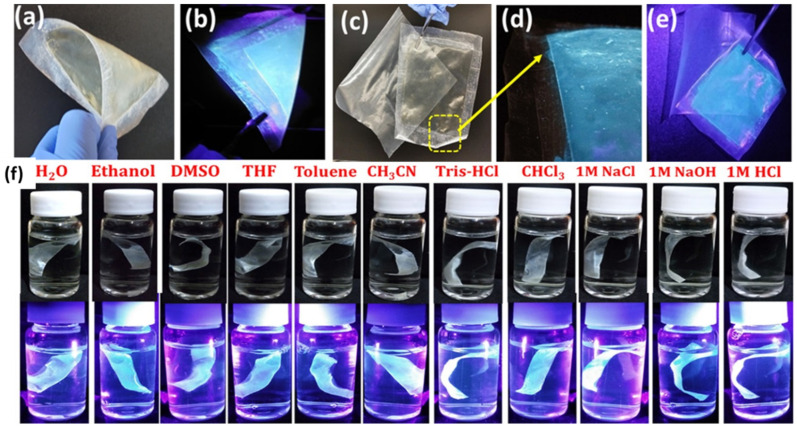
CDs-coated polypropylene film twisted in (**a**) daylight and (**b**) under UV light (**c**), comparative images of coated and noncoated films, (**d**) magnified image of (**c**,**e**) coated and noncoated films under UV irradiation, (**f**) coated films in different solvents and their daylight and UV irradiated images. Reproduced with permission from ref. [17].

Wu et al. illustrated the incorporation of quaternary CDs into poly(ethylene glycol) to make PEG/CDs composite solid films [62]. These films have strong and tunable blue–red emission and a quantum yield of 12.6%, which is comparable to liquid CDs. The hydroxyl functionalities of PEG can remove nonradiative defects/surface states to further passivate CDs. In theory, the bonding of PEG through an alkoxy linkage involves a strong surface modification, which offers an ascent to a quasi-continuous band in the conduction band. This band substitutes isolated levels in CDs that are not passivated enough to offer tunable emission. In a report, Das et al. reported the immobilization of heteroatom-doped CDs onto nonpolar plastic films (polypropylene) by means of photochemical covalent grafting in order to enhance the storability of food products without impacting the quality of plastic films [17]. The CDs-coated polypropylene film showed antioxidant ability and antifogging properties and was used for the packaging of fruit. The photoluminescent property and durability (Figure 3) was evaluated against different simulated drastic environmental conditions to ensure its application in real life. Karak et al. developed a straightforward eco-friendly technique to fabricate hyperbranched polyester/CDs nanocomposites by in situ polymerization [63].

The thermosets were synthesized by curing polyester nanocomposites with glycerol-based hyperbranched epoxy and fatty-acid-based poly(amido amine) hardener. This resulted in the formation of the thermosets. For the thermoset nanocomposites, considerable improvements in mechanical parameters such as tensile strength (7.8–47 MPa), Young’s modulus (243–745 MPa), toughness (17.82–51.1 MJ m^−3^), and scratch hardness (4–10 kg) were observed. A thermogravimetric study validated the high thermostability (234–265 °C), and differential scanning calorimetry confirmed a glass transition temperature in the range of 49–56 °C.

**Figure 4:**
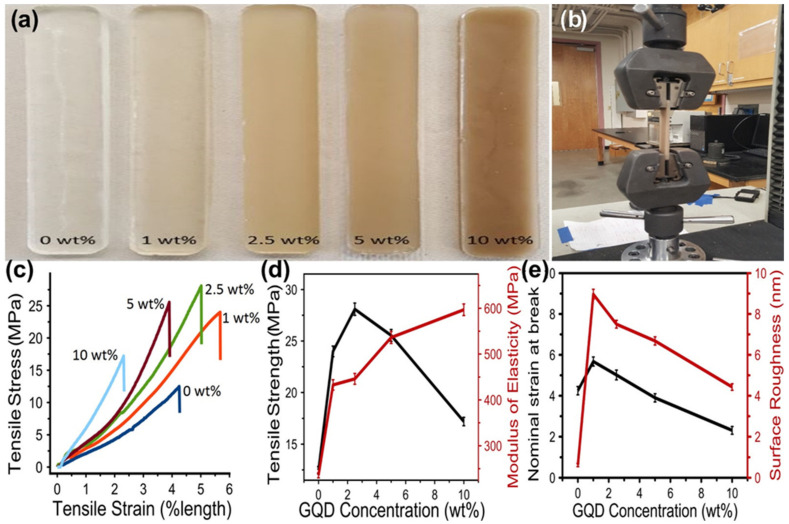
(**a**) Digital images of epoxy-GQD nanocomposites with various loadings of GQDs. (**b**) Instron setup for tensile test. (**c**) Stress–strain curves of various nanocomposites. (**d**) Tensile strength and modulus of elasticity against GQD concentrations, showing that the addition of GQDs made the epoxy stiffer and stronger. (**e**) Tensile strain and surface roughness of composites. Reproduced with permission from ref. [66].

Gogoi and coworkers reported in situ and ex situ synthesis of tannic-acid-based waterborne hyperbranched polyurethane (WPU) nanocomposites as luminescent and biocompatible composites by incorporating various weight percentages of CDs from corms of *Colocasia esculenta* [64]. The thermosets exhibited a good fluorescent activity, which served as confirmation of the polymer matrix’s efficacy in preventing solid-state quenching. The thermosetting nanocomposites demonstrated a dose-dependent improvement in tensile strength > 4.6-fold, toughness > 4.2-fold, and impact resistance > 1.25-fold compared to pristine WPU. The thermosetting films showed excellent adhesion, proliferation, and differentiation of MG 63 osteoblast cells in vitro. Using a coordination-driven self-assembly approach, a new sensor based on Ag_2_S/chitosan films was successfully constructed by Yue et al. [65]. Experiments certainly proved that Ag_2_S had no influence whatsoever on chitosan film. FTIR, DSC, SEM, and fluorescence analysis were used to conduct investigations on the structural and sensing properties of the nanocomposites. Erogbogbo reported that infusing GQDs into an epoxy polymer matrix (Figure 4a) results in a more than 2-fold increase in toughness, and tensile strength compared to its original tensile strength, and a 2.5% times increment in Young’s modulus compared to the neat polymer resin without GQDs [66]. Figure 4b shows the Instron setup for the tensile test. Figure 4c shows that introduction of GQDs to the epoxy made it stronger initially, but after 2.5 wt%, the strength began to drop. The relationship of tensile strength and modulus of elasticity vs. different concentrations of GQD showed (Figure 4d) that the addition of GQDs made the epoxy stiffer and stronger. The tensile strain and surface roughness of nanocomposites are depicted in Figure 4e. The authors suggested that GQDs that include both acid and alcohol functionalities can facilitate large loading percentages, which, in turn, result in composite materials that are concurrently more robust and more abrasion-resistant.

The very high surface-to-volume ratio of quantum dots contributes to their surprising and novel physical features. If these materials were to be utilized in photovoltaic applications, the anti-factors in the classical theoretical constraints on the efficiency of a solar cell mentioned in the Shockley–Queisser thorough balanced analysis would be eliminated due to their quantum-confinement-related features. Through multi-exciton production, quantum dots are predicted to increase solar efficiency beyond that of traditional solar cells (MEG). One of the most appealing features of quantum dots is their tunable band gap as a function of size. Electrospinning and the consecutive ionic layer adsorption and reaction approach were used to create cheap quantum dot solar cells, as described by Unni et al. [67]. CdCl_2_ and Na_2_Se are used to deposit CdSe nanocrystals on SnO_2_ nanofibers. Electrospinning was used to successfully create a CdTe quantum dot/PVA (poly vinyl alcohol) composite nanofiber. PVA stands for polyvinyl alcohol [68]. Over the course of the past decade, optical nanofibers have demonstrated potential new directions that may be taken in quantum photonics. Several theoretical approaches for interacting neutral atoms with nanofibers, such as entrapping atoms around a nanofiber and efficiently channeling single-atom emission into the nanofiber-guided modes, were described by Kien et al. between 2004 and 2007 [69]. Homogeneous morphology is one of the hallmarks of the polyacrylonitrile (PAN) fibre generated using the supersaturated recrystallization and electrospinning method, which is based on the inorganic dual-phase CsPbBr_3_-Cs_4_PbBr_6_ quantum dots (CPB QDs) [70]. Based on the trace-recording capability of CPB@PAN fibre, our experimental and theoretical results presented a simple technique for developing biological protection display, biotic detection, and moisture-proof forewarning.

## 5. Optical Properties of Quantum Dots/Polymer Composites

Quantum dots, as was previously noted, have a unique optical feature; nonetheless, the vast majority of quantum dots do not exhibit any substantial absorption in the visible area, but instead display a wide spectrum of fluorescence emission. They display multicolor emission when activated at the suitable frequency and produce light of varying wavelengths. They emit light that ranges from blue to near infrared in the visible spectrum. After the production of the composite with the polymer, it has been demonstrated that the fluorescence characteristics of the dots may be maintained in their original state [71,72]. Carbon, oxygen, and certain heteroatoms including nitrogen, boron, sulfur, silicon, and phosphorus are the most prevalent elements found on compact discs (CDs). The vast majority of CDs exhibit no substantial absorption in the visible spectrum, although they do produce a spectrum of colors ranging from blue to red. In this article, we talk about the optical features of CDs/polymer composites, including the UV–vis absorption qualities and FL properties. Hu et al. were successful in producing CDs/polymer composites with FL characteristics. It is possible for the polymer system to maintain the FL features of the original CDs [73]. It has high viscosity and can be used to generate FL ink that may be used for different designs, and it presents vivid solid-state FL at room temperature. There are just a few carbon dots/polymer composites that have an intriguing UV–vis absorption behavior. The quantum dots are transplanted onto the composite without causing any change in the carbon dots’ innate optical capabilities, and the composite serves to protect the carbon dots’ fluorescence qualities. The PL intensity of the composites shows a linear rise in conjunction with an increase in the concentration of the carbon dots. The optical characteristics of a variety of quantum dots and polymer composites are compared and contrasted in Table 1. In spite of the fact that the quantum yields of the quantum dots/polymer nanocomposite are rarely addressed in the research literature, the values are, on average, rather considerable. Because the optical properties of composites are entirely dependent on the optical properties of the quantum dots, the composite material exhibits vibrant colors such as green, blue, cyan, red, yellow, and orange. These colors are determined by the fluorescence emission wavelengths of the quantum dots that make up the composite. In addition, the physiochemical characteristics of the quantum dots have an impact on the optical properties of the carbon dot/polymer composite material. Practically speaking, the diameter of a carbon dot can range from 1 to 100 nm, but in practice, the typical size of these quantum dots is 10 nm, with a few bigger particles having a size of 60 nm [30]. When the size of the quantum dot is reduced to less than its Bohr excitation radius, a phenomena that is also known as the quantum confinement effect takes place. This leads to the creation of quantum dots of a higher quality [74]. As a consequence of the quantum confinement effect, the band gap of these particles varies depending on their size, which leads to a number of optical features that are size-dependent. CD fluorescence is difficult to characterize because it is wavelength- and solvent-dependent. It is well known that a redshift in the excitation wavelength will cause a redshift in the emission spectrum, which will then give birth to the red edge excitation shift. This phenomenon has been linked by some writers to the quantum confinement effect, which is analogous to the situation involving semiconductor nanoparticles. This effect suggests that a heterogeneous core size distribution is responsible for a variety of various emission wavelengths [75]. However, there are many who choose to understand it in terms of the fluorophore emission that occurs on the surface [76]. In point of fact, solvatochromism in CDs has been described by a number of different researchers [77]. The p-phenylenediamine is subjected to a simple one-step solvothermal treatment in order to produce one variety of amphipathic CDs [78]. When the as-prepared CDs are dissolved in various solvents, one may see a robust solvatochromic activity with emission that is adjustable from blue to green and exhibits a tight excitation-independent emission characteristic. The fluorescence property, on the other hand, is determined by the functional group as well as the stiffness of the core structure. Quantum dots that include carboxyl or amide groups glow green, carbon dots that have hydroxyl groups fluoresce blue, and replacing hydroxide groups with amine groups induces a red shift in fluorescence, as has been seen.

## 6. Sensing Application of Quantum/Polymer Composite

### 6.1. Detection of Biologically Important Molecules and Disease Biomarkers

**Figure 5:**
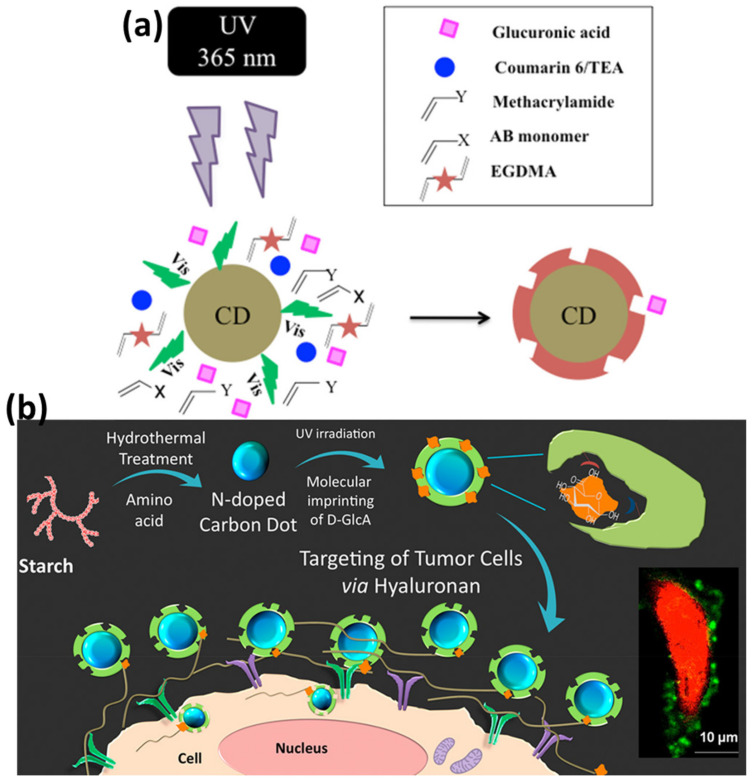
(**a**) CDs triggers the photopolymerization of MIP shell. (**b**) Schematic representation of core–shell CD-MIPs for biotargeting of tumor cells via hyaluronan. Reproduced with permission from ref. [93] © 2023 American Chemical Society.

Caffeic acid has unique properties such as anti-inflammatory, antitumor, and immunomodulatory. In modern clinical practices, caffeic acid is frequently employed in the treatment of leukopenia, thrombocytopenia, and hemostasis, among other applications [97]. Hu et al. proposed CDs coated with MIPs based on a novel and highly sensitive technique for determining caffeic acid by a one-pot sol–gel method [89]. This platform took advantage of both the good sensitivity of CDs and the high selectivity of MIPs and showed great selectivity and affinity for caffeic acid. The intensity of the fluorescence signal by CDs@MIPs was found to decrease in a linear fashion with increasing concentrations of caffeic acid (range: 0.5–200 M) and the detection limit was identified to be 0.11 µM. Furthermore, caffeic acid was effectively detected in human plasma using the proposed approach. For the objective of targeted fluorescence imaging of cervical cancer, a CDs-embedded epitope imprinted polymer (C-MIP) was developed by Zhang and coworkers [98]. This approach utilized CDs as carriers, acrylamide as the major functional monomer, and N-terminal EGFR nonapeptides as templates. This platform was designed to recognize the epidermal growth factor receptor in a very specific manner (EGFR). The fluorescence response assays demonstrated that C-MIP possessed both high levels of sensitivity as well as excellent selectivity when it comes to the epitopes of EGFR. The linear range of fluorescence quenching was detected in the region from 2.0 to 15.0 µg/mL, and 0.73 µg/mL is the lowest amount that can be used to measure it. Moreover, targeted fluorescence imaging in vitro and in vivo demonstrated the capability to specifically detect EGFR [21]. Zong et al. developed ternary nanocomposite based on MOF-derived zinc oxide nanopolyhedra/GQDs/polyaniline via an in situ polymerization technique [99]. A flexible PET substrate with interdigital electrodes was used to make the nanocomposite. At ambient temperature, the gas-sensing capabilities of the sensors were tested by exposing them to varying amounts of acetone in a controlled environment. The experimental findings of this sensor revealed high response, strong repeatability, great stability, excellent selectivity, and rapid response or recovery features in response to low concentrations of acetone gas. Attributed to the synergistic effect and heterojunction generated in the nanocomposite, this sensor was reported to be a good candidate for detecting ppb-level acetone at room temperature. Cholesterol is a kind of lipid that plays an important role in the maintenance of the fluidity and structural integrity of cell membranes [100]. However, an elevated level of cholesterol in the serum has been shown to reduce blood flow, which can lead to a number of cardiovascular problems [101]. Since the detection of cholesterol is considered to be very significant in medical laboratories, this molecule was selected as the target molecule by several researchers. In a report, Hu et al. presented an approach that combines CDs doping with polymer encapsulation in order to develop a stable perovskite CH_3_NH_3_PbI_3_-based photoelectrochemical platform for cholesterol sensing [102]. The sensing probe was based on photoelectrochemical reactions. The water resistance was significantly increased due to the addition of a PVDF protective coating. Because of the functional carbonyl groups and hydroxyl groups that CDs possess, the perovskite grain boundaries were successfully passivated using these very inexpensive and easily accessible nanomaterials. A straightforward thermal polymerization technique, followed by elution with hexane, was used to anchor MIP onto PVDF-CH_3_NH_3_PbI_3_@CDs. The detection limit for cholesterol was found to be 2.1 × 10^−14^ mol/L, which is lower than the majority of the other techniques currently used to detect cholesterol. In the ongoing battle against cancer, the development of novel affinity techniques that can selectively detect, locate, and quantify biomarkers for diagnosis or prognosis is essential. Recent developments in the fields of glycobiology and cancer research have led to the discovery that an accumulation of hyaluronan is closely connected to the growth of tumors in patients [103,104,105]. In a report, Haupt and coworkers coupled CDs with MIPs to develop a sustainable advanced optical tool for investigating cancer biomarkers [93]. CDs were synthesized from starch and l-tryptophan using the hydrothermal synthesis. The MIP was prepared directly as a thin layer on the CDs by utilizing the photoluminescence of CDs to trigger localized photopolymerization under UV irradiation (Figure 5a). The initiator for the polymerization process was coumarin 6/TEA; functional monomers used were AB and methacrylamide; and the cross-linker was EGDMA. The MIP shell played the role of the specific recognition of glucuronic acid, which is a substructure (epitope) of hyaluronan. Core–shell CD-MIPs were then used for the biotargeting and cell imaging of hyaluronan in HaCaT cells, as well as in the HeLa cell line, which served as models for healthy cells and transformed cells, respectively (Figure 5b).

### 6.2. Sensing of Toxic Pollutants

The fast expansion of industrialization has resulted in the production of a wide variety of pollutants as by-products, which have been released into the air–water ecosystem. Toxic contaminants in water pollution are one of the most serious problems facing the environment today.

**Figure 6:**
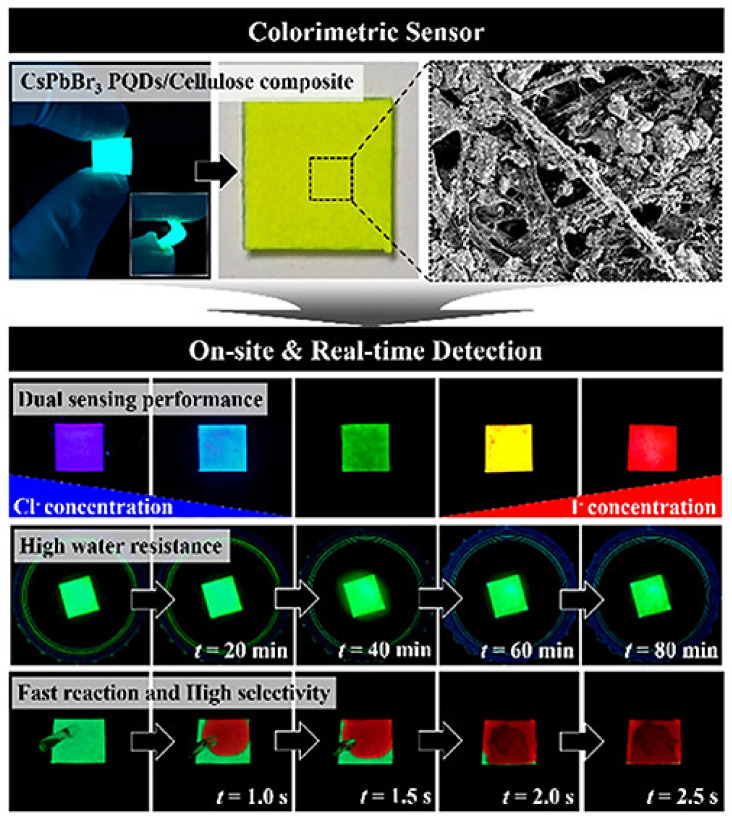
Cellulose-based colorimetric sensor coupled with CsPbBr_3_ PQDs. Real-time detection of Cl^−^ ions and I^−^ ions by naked eye. Reproduced with permission from ref. [109] © 2023 American Chemical Society.

There is a growing focus on molecules that have an adverse effect on the environment or public health. It is the goal of scientists to build sensors that can detect these pollutants at the lowest feasible level, so that they can be detected even before they begin to cause serious damage [52,106,107]. Jiang et al. constructed thin film nanocomposite membranes integrating GQDs with improved water permeability and antifouling performance [108]. As the GQDs’ concentration increased, the surface roughness of the membranes reduced and their surface hydrophilicity increased. GQDs were easily incorporated into a polyamide layer during the interfacial polymerization of piperazine and trimesoyl chloride. According to the results of nanofiltration (NF) investigations, the greatest water flow of the TFN membranes was up to 102.0 liters/square meter per hour with an operating pressure of 0.2 MPa. This water flux was about 6.8 times higher than that of a pristine polyamide membrane. In another report, Huh et al. presented a facile and portable cellulose-based colorimetric sensor coupled with CsPbBr_3_ perovskite quantum dots (PQDs) for the quick detection of chlorine and iodine ions using just the naked eye (Figure 6) [109]. Along with having great photoluminescence capabilities, the composite demonstrated high stability and endurance when exposed to a wide range of humidity conditions. To develop the composite, a hot injection approach was used, which allowed the deposition of PQDs into the porous cellulose fibers during the synthesis process. The change in color was caused by the anionic exchange between the halide ions, which controls the band gap of the QDs.

Within 5 s, the visible color changed from blue to orange as an ion exchange took place, and a change in the photoluminescence property was noticed at the same time. The PQDs/cellulose composite exhibited outstanding sensitivity toward the detection of I^−^ ions ranging from 0.1 mM to 1 M. The LODs for I^−^ ions and Cl^−^ ions were 2.56 mM and 4.11 mM, respectively.

**Figure 7:**
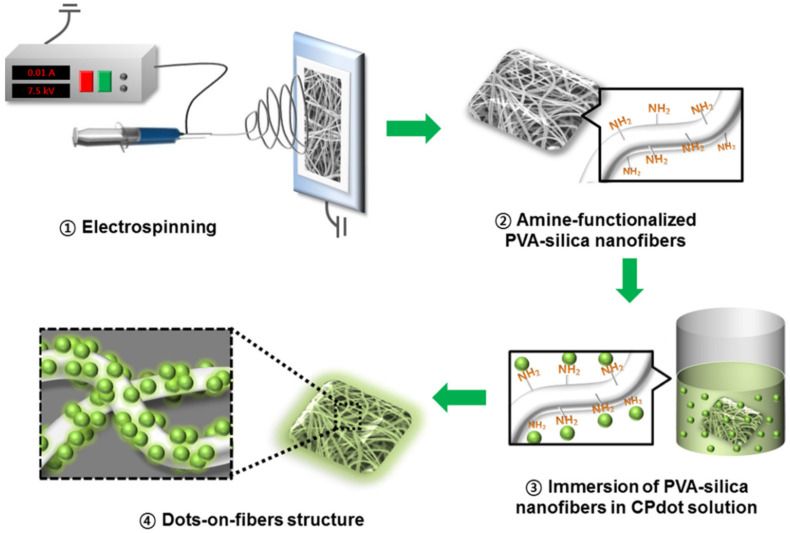
Schematic presentation for the development of dots-on-fibers structure. Reproduced with permission from ref. [110] © 2023 American Chemical Society.

For practical purposes, it is vital to be able to quickly and selectively detect chemical warfare agent simulators. In a report, a unique chemical warfare agent sensor was fabricated (Figure 7) with a dots-on-fibers hybrid nanostructure by using a facile electrospinning technique and subsequent immobilization processes by Lee et al. [110]. This sensor system was based on conjugated polymer dots, which were immobilized on the surface of PVA–silica nanofibers. It was found that CP dots and amine-functionalized electrospun PVA–silica nanofibers interacted with one another by means of an electrostatic interaction, and this interaction was able to remain stable over a wide range of continuous mechanical forces. By evaluating the QY, it was possible to validate the significant increase in the fluorescence intensity of the dots-on-fibers, which supported the prospect of their usage as highly sensitive nanocomposites for applications involving sensing. Because of the transfer of electrons between the quinoxaline unit in the polymer and the organophosphorus simulant, the developed fluorescent DoF hybrid was quenched with the occurrence of a chemical warfare agent simulant. The detection time was almost immediate, and a very low limit of detection was obtained (1.25 × 10^–6^ M). In addition, the selectivity of the detection was demonstrated over other organophosphorus compounds.

### 6.3. Quantum Dots Confined 3D Soft Matrices-Based Sensors

The creation of cutting-edge materials is dependent on discovering novel ways to combine two materials that could not be more unlike one another. It has been demonstrated that loading quantum dots (QDs) into a three-dimensional (3D) network of hydrogels is a successful technique for improving the synergy between the various components. The novel QDs/hydrogel composites that were created as a consequence have a broad variety of applications in several critical domains such as the environment, energy, and health. The derivatization sequence, which is seen from a somewhat more practical standpoint, is used to categorize synthetic techniques into a variety of various categories. These categories are then categorized according to the QDs and hydrogel.

**Figure 8:**
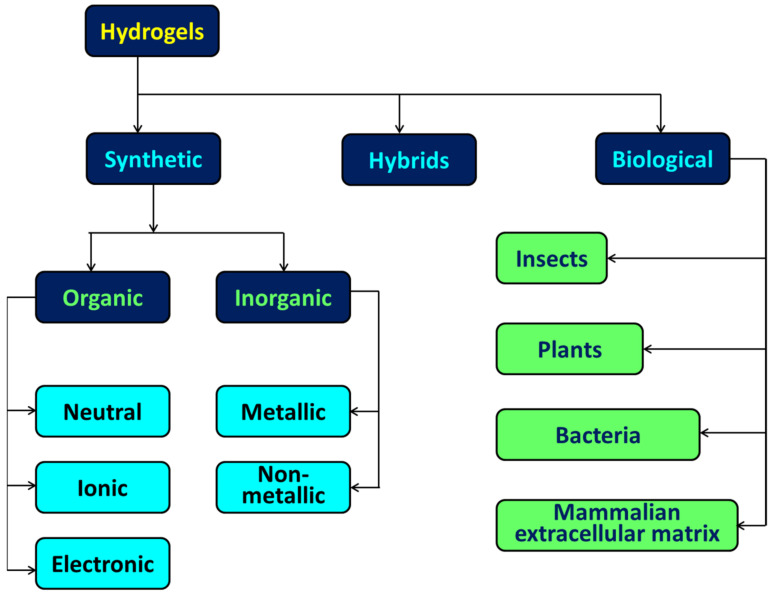
The categorization of hydrogels with respect to their constituents and their origins.

The cross-linking of hydrophilic polymer chains results in the formation of a three-dimensional (3D) network colloidal gel known as hydrogel. The content and origin of hydrogels are two of the primary factors that determine whether they are categorized as biological, synthetic, or hybrid. The mechanical characteristics of biological hydrogels are inferior to those of synthetic hydrogels because biological hydrogels are made up of natural polymers, which are mostly polysaccharides or proteins. Hybrid hydrogels, on the other hand, can be improved by incorporating the former into synthetic hydrogels. Figure 8 provides further information. The cross-linking of the polymer chains prevents the entire system from becoming soluble, despite the strong attraction of the polymer chains for water. Various cross-linking procedures make it feasible to make further adjustments to the structure and characteristics, which in turn makes it possible to develop hydrogels with very particular functionalities. Therefore, hydrogels may also be classified according to the sort of cross-linking that occurs. In most cases, the production of hydrogels that are physically cross-linked takes place either through the formation of noncovalent bonds between polymer chains or by the formation of entanglements within the polymer network. On the other hand, hydrogels that have been chemically cross-linked have a greater mechanical strength. Because hydrogels are so good at retaining water and possessing outstanding mechanical qualities, they have major uses in a variety of fields, including tissue engineering, medication delivery, and the treatment of wastewater, among others. When bulk materials are reduced to the form of nanoscale particles, there is potential for a number of their physical characteristics to undergo significant shifts. Many optical nanomaterials that have the capacity to absorb light and convert it into other forms have emerged as hot subjects in the disciplines of biological imaging, diagnostics, and sensing as a direct result of the growth of chemistry and nanotechnology.

**Figure 9:**
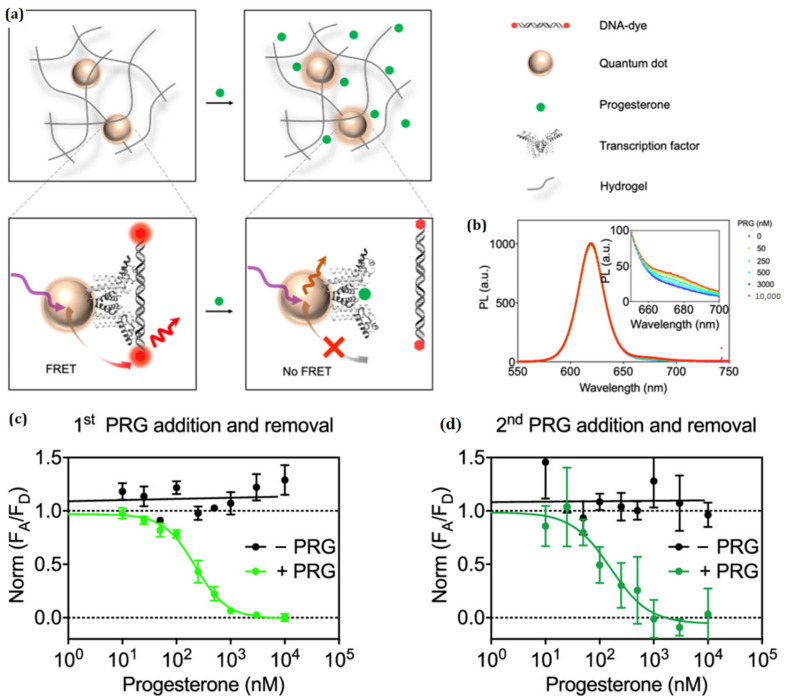
(**a**) FRET sensor that is based on QDs and is immobilized in the hydrogel. An image of a schematic depicting the diffusion of progesterone into the hydrogel and FRET-based sensor utilizing the TF–DNA binding mechanism, (**b**) Representative spectra of sensor response to progesterone. (**c**) the first cycle of progesterone (PRG) exposure and removal, the sensor exhibited a response. (**d**) The second cycle of progesterone exposure and removal elicits a sensor response when exposed to progesterone on a regular basis. Reproduced with permission from ref. [117] © 2023 American Chemical Society.

Since Brus first put up the idea of colloidal QDs in 1983, researchers have been hard at work developing a variety of different QDs synthesis processes [111]. The research that has been conducted thus far on QDs-based hydrogels is considerable, and the majority of it has focused on their usage in sensing applications. Hydrogel sensors based on QDs have the ability to transform chemical information into signals that are relevant for analysis, allowing for the early identification and precise assessment of target analytes. This is made possible by the distinctive optical and electrical characteristics of QDs [112]. The vast majority of QDs-based hydrogels function primarily as fluorescence sensors that are driven by the idea of optical signal propagation. When demonstrating the accurate results using a number of different fluorescence parameters, the luminous intensity is typically the one that is selected. As a result, the term “target analytes” can refer to a wide variety of the elements that impact the luminous intensity of the QDs. Recent studies have shown that fluorescent QDs-based hydrogels can reliably detect several heavy metal ions [113,114,115,116]. Their practical implementation is severely hindered, however, by drawbacks such as costly and accurate instruments, complex sample-handling procedures, and time-consuming detection periods. As a result, a QDs-based hydrogel sensing system has the potential to compensate, at least partially, for the shortcomings of conventional ion-sensing technologies. The life sciences, including diagnostics and medical equipment, are benefiting tremendously from the transformation brought about by digital health. Portable advanced technologies will further fuel the growth of digital health and will present a multitude of options for diagnosis and monitoring throughout the clinical landscape. These possibilities will cover a wide range of conditions, from malignancy and trauma to addictive behavior. It is essential for further device fabrication and the integration of wearable technology for immunosensors to be immobilized in or on a functional material before proceeding. As the first move toward the implementation of such a device, Chen et al. described the construction and evaluation of an immobile quantum dot–transcription factor–nucleic acid combination for progesterone detection (Figure 9). This complex was designed to detect the hormone progesterone [117]. The immobilization matrix consists of a hydrogel, and the sensor, which is made up of a polyhistidine-tagged transcription factor connected to a quantum dot and a fluorophore-modified cognate DNA, is encased within the hydrogel. In addition to being transparent to visible light, delicate, and stretchy, the hydrogel also has the ability to ensnare the quantum dot–transcription factor DNA assemblage while yet allowing the analyte, progesterone, to flow through freely.

**Figure 10:**
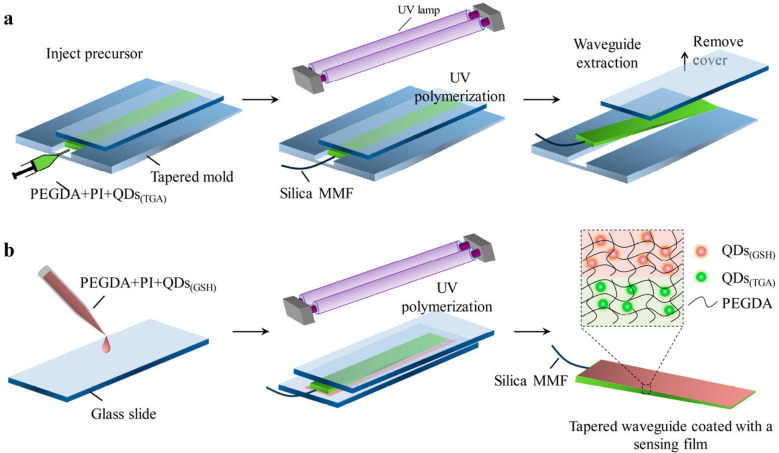
(**a**) Creating the tapered hydrogel waveguide through manufacturing. In situ photoinduced cross-linking of hydrogel precursor is performed in a tapered rectangular mold in order to create the waveguide taper. (**b**) Film coating. A thin film coating is formed through polymerization of a thin layer of hydrogel precursor on the waveguide surface, for which the waveguide taper is clamped between two microscopic glass slides to achieve a uniform thickness. Reproduced with permission from ref. [119] © 2023 American Chemical Society.

The sensing that can be conducted using hydrogels containing fluorescence QDs is based on the sensitivity of the PL of QDs to the surface state of the QDs. For a few instances, the objective substances destabilize the hydrogel system by interacting with the physiochemical bonds that are responsible for maintaining the three-dimensional structure. This, in turn, causes accumulation quenching of QDs, which enables the analyte identification to be accomplished by the use of this sensing sequence. The creation of soft and implantable optical waveguides by employing polymer hydrogels such as polyacrylamide, poly(ethylene glycol) diacrylate, and agarose has generated widespread interest in biosensing and photomedicines [118]. In contrast to its desirable mobility and cytocompatibility, hydrogel optical waveguides offer continuous monitoring without contaminating the samples being tested. This is only one of their many benefits. The fabrication of a hydrogel-based waveguide platform that is capable of in situ sensing in a variety of settings, as well as the continuous monitoring of metal ions with a rapid response, is of the utmost importance. In their study, Guo et al. presented the design and manufacturing of a tapered hydrogel waveguide that was covered with a thin sensing layer (Figure 10). This waveguide was used for ratiometric and in situ detection of metal ions with a quick response [119].

**Figure 11:**
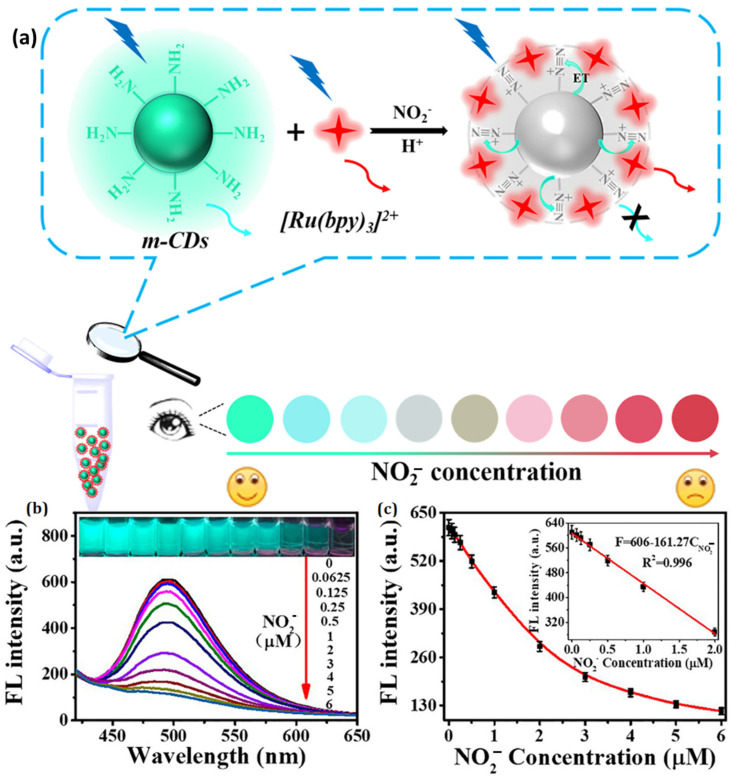
(**a**) Visual NO_2_^–^ detection with m-CDs@ [Ru(bpy)_3_] Ratiometric probe. (**b**) Fluorescence spectra of m-CDs after adding NO_2_^–^ (**c**) m-CD fluorescence vs. NO_2_^–^ concentration. Reproduced with permission from ref. [124] © 2023 American Chemical Society.

In the case of direct assessments, the fluorescence signal that is produced by the analyte of interest is detected at a signal intensity that is proportional to the analyte concentration [120,121,122]. The analyte of interest diffuses into the three-dimensional pores of the hydrogel and forms particular chemical interactions with the functional groups that are located on the surface of the QDs. Through the specific sensing mechanism, this causes a rise or decrease in the luminous intensity of the QDs [114,123,124,125]. One-step hydrothermal carbonization was used by Zhan et al. to produce carbon dots (m-CDs) from m-phenylenediamine (mPD). NO_2_^−^ reacted with m-CDs in the presence of high acidity (Figure 11), and the diazo reaction occurred [124]. When there was a higher concentration of NO_2_^−^ in the air, a fascinating phenomenon occurred: the fluorescent color of the ratiometric probe changed from cyan to red as the level of NO_2_^−^ rose. On the basis of these results, a ratiometric fluorescent-based portable agarose hydrogel test kit was created, and it was utilized for on-the-spot measurement of NO_2_^−^ concentration within ten minutes. In a situation that might be described as indirect, the concentration of the target analyte is perceived to have been achieved through the inhibition of the production of secondary compounds. When QDs-based hydrogels were utilized, for instance, to detect bacteria or glucose, the intended substance had an influence on the luminescent intensity of the QDs through the employment of enzymes rather than a close coordination [126,127]. Many different tests, analyses, and eliminations must be performed before a final decision can be made on which QDs-based hydrogel fluorescence sensor is the best for a certain analyte and QDs-based hydrogel fluorescence sensor [128].

Several more analyte-independent factors, including sample matrix light scattering, device performance, and probe concentration, all influence the fluorescence state of quantum dots (QDs) used in detecting probes. As a result, these QDs must be distributed in the primary analysis prior to fluorescence quantification. These characteristics will most definitely provide challenges in terms of obtaining precise and reliable assessments. The adjustment of probe intensity is one of the more time-consuming steps, as it is necessary not only to achieve optimal QD fluorescence intensity but also to avoid flaws caused by NP agglomeration or biochemical decomposition. Fast responsiveness, dependability, repeatability, and stability are all essentials when it comes to sensor design [129]. Xu et al. demonstrated that the prepared flavonoid-derived CDs/CS hydrogels were significantly more sensitive to detect Pb^2+^ than the electrochemical and fluorescent sensors that were reported at the time. These hydrogels even had comparable sensitivity to signal-amplified electrochemical aptosensors [113]. When compared to a solution sensor that is based on QDs, hydrogels offer a number of distinct benefits to QDs sensing systems. These benefits include a high level of stability, a rapid response, a geometry that can be adjusted, manageability, renewability, and diversified outcome acquisition, among others.

As a result of the renewable, nontoxic, cost-effective, and biocompatible properties that they possess, there has been some effort made to utilize a wide variety of easily accessible natural biomass and biowaste as precursors. These natural biomass and biowaste sources include things such as vegetables, fruits, milk, and even waste [130]. It is possible to significantly boost the fluorescence activity of CQDs by further doping of heteroatoms into the carbonaceous framework as well as through surface passivation. According to previous research, carbon quantum dots (CQDs) may be sustainably synthesized from banana peel waste using a straightforward hydrothermal technique [131]. By adjusting the excitation wavelength, the CQDs were able to effectively inject their stain throughout the nematode’s whole body, illuminating the colors in brilliant detail. QDs have the potential to increase the utility of semiconductor nanocrystals in sensorics by being utilized in considerably more analyte specific FRET (Förster resonance energy transfer)-based sensors. Numerous quantum dot FRET applications have emerged, as have methods to boost energy transfer. To examine protein-binding locations, FRET experiments were conducted, with the acceptor dyes attached to the protein and allowing FRET when the assembly was adsorbing on the QD surface [132]. However, diagnostic strategies employing modified quantum dots depend more on impermeable polymer shells and good physical and biological shielding of the quantum dot to avoid nonspecific adsorption of proteins and rapid degradation resulting in a fluorescence loss. The in vivo imaging technique used here offers a noninvasive method of detecting deep tissue areas in mice and even bigger species with great sensitivity and contrast, without the need for radioactive radiation or bulky equipment setups. The in vivo targeting of tumor cells with antibodies directed against Her2 markers is another intriguing area of use [133]. Self-illuminating quantum dots are another development that has helped advance the field of in vivo imaging. These polymer-coated and luciferase-modified nanocrystals do not require any other light source in order to be excited. Through a process called bioluminescence resonance energy transfer, this photon energy makes the quantum dot excited (BRET). When using this excitation technique, practically all of the autofluorescence is removed; nevertheless, the released photons can still be absorbed or scattered, which requires sensitive detection [134,135,136]. Due to the ease of access from the outside of the cells and the unneeded transit through the cell membrane, much of the research in this area has concentrated on membrane-specific markers. The approach of internalizing QDs makes it possible to locate subcellular cavities such as mitochondria or the nucleolus by using specific targeting peptides. This enables the marking of the subcellular compartments in living cells, which is necessary for the study of cellular biology in the future [137].

## 7. Summary and Future Perspectives of Polymer/Quantum Dots Composites

It has been established that nanocomposites made of biocompatible polymers and quantum dots have a substantial amount of potential that has not yet been exploited in the fields of biological and medical application [138,139]. When it comes to the process of fine-tuning the surface features of QDs, conferring colloidal persistence, and introducing functionality into the QD core for end-use applications, a polymer shell is a key component. The generation of various types of biocompatible polymer/QDs systems based on related usage has seen tremendous progress among chemists and has resulted in a number of remarkable achievements. In spite of such advancements, obstacles still exist for biomaterials and quantum dots when they are used in biomedical activities. Furthermore, as this article mentions, the fields of biocompatible polymers and quantum dots detectors and sensors have room for further development and might be better used.

To begin, the functions that interconnect surfaces that perform in establishing the material characteristics of polymer/QDs hybrids are quite essential. During the manufacturing procedures involving polymers and quantum dots (QDs), the polymers engage with and substantially modify the interface of the basic QDs. This results in the introduction of a “defect” that thus diminishes the extremely high quantum yields of the as-developed QDs. As a result, there is an immediate need to design polymers, manage the production strategy, and reduce “defect” locations on QDs in order to produce highly luminous ultrabright polymer/QDs probes. Professionals are guided to manufacture sustainable polymer/QDs probes with extremely high fluorescence by having a technical understanding of the interaction of polymers with the surfaces of metallic QDs, which undoubtedly persists as a significant topic in the polymer/QDs sector.

Secondly, the diameter of the polymer or QDs sensor ought to be sufficiently tiny so that cells may endocytose it with relative ease. When contrasted to the hydrodynamic dimensions of as-prepared QDs, the hydrodynamic diameters of polymer/QDs typically rise by 20–50 nm. In order to achieve extremely effective endocytosis, the sizes of the polymer or QDs need to be reduced while yet maintaining their physical stability. On the other side, size-minimized polymer/QD hybrids provide a unique chance for biochemical engineering and intracellular monitoring at the level of a single molecule [74]. Since this is the case, the next challenge that will need to be solved is figuring out how to make reduced polymer/QDs probes that can be used in a range of bioapplications. When it comes to decreasing the size of polymer/QDs probes, having access to a comprehensive range of high-quality polymers will be absolutely necessary. Colloidal stability of the polymer and QDs should be preserved throughout a wide range of in vivo biological conditions, including supersaturated electrolyte solutions, varying temperature ranges, and a broad spectrum of pH values. To be more precise, the polymer layering must limit undesired protein binding on QDs as well as the self-aggregation of QDs, both of which have an effect on the luminous effectiveness of the polymer/QDs probe. This necessitates the development of typically more multifaceted polymer/QDs complexes by scientists in order to ease the usage of QDs for a longer period of time in environments that are devoid of agglomeration.

Even though polymer coatings make QDs more stable and reduce their toxicity, we do not want the combination of polymer and QDs to remain in the bloodstream for an extended period of time after they have been identified and photographed. This is because we do not want the combination of polymer and QDs to be toxic. The removal of polymer or quantum dots from the body continues to be a key barrier for applications involving these nanomaterials. Knowing how polymer/QDs pass through the system will create a landmark in this sector and establish the criteria for the in vivo bioapplication of polymer/QDs hybrid nanomaterials, despite the fact that polymer/QDs have achieved accomplishment and are extensively used in vitro.

## Figures and Tables

**Table 1:** The fluorescence (FL) and ultraviolet–visible (UV–vis) absorption characteristics of a number of different QD/polymer composites.

Surface-Decorated Polymers/Moieties	Excitation Wavelength (nm)	Emission Wavelength (nm)	Quantum Yield (%)	Absorbance (nm)	Emission Color	Refs.
MIPs	370	470	51.8	370	Blue	[79]
Chitosan/PVA	360	436	N/A	360	Cyan	[80]
PVA	360	~470	47	294/340	Blue	[81]
Polyethylene glycol	455	550	14.86	200–500	Yellow	[82]
Magnetite-MIPs	370	470	N/A	N/A	Blue	[83]
Cu-alginate	400	513	N/A	N/A	Green	[84]
C-dots/PVB film	400	550	N/A	353, 410, 500	Green–Blue, Orange–Red	[85]
CD polymer	455	550	14.86	200–500	Yellow	[86]
CDs@silica@MIPs	380	>450	N/A	288	N/A	[87]
WCDs@polystyrene	380	~590	10.7, 15.2	N/A	Orange and Blue	[88]
CDs@MIPs	360	450	N/A	250–300	Blue	[89]
Polyethyleneimine	470	~565	1.9–4	290, 340, 380	Cyan	[90]
CDs@PVA	365	420–440	N/A	~350	Blue	[91]
C-dots/PVA	360	459	8.64	282, 341	Green	[92]
CD-MIP glucuronic acid	445	~500	0.97	~350	Blue	[93]
Poly(VPBA-AAm)	900	515	N/A	241	Blue	[94]
PAN nanofibers	350, 477, 530	560, 598, 660	N/A	314, 316, 318	Red, Green, Blue	[95]
PVA	390	~460	44	286, 355	Cyan	[96]

## Data Availability

Not applicable.
